# Neural and behavioral effects of modification of visual attention in body dysmorphic disorder

**DOI:** 10.1038/s41398-022-02099-2

**Published:** 2022-08-10

**Authors:** Wan-Wa Wong, D. Rangaprakash, Joel P. Diaz-Fong, Natalie M. Rotstein, Gerhard S. Hellemann, Jamie D. Feusner

**Affiliations:** 1 Centre for Addiction and Mental Health, Toronto, ON Canada; 2 Athinoula A. Martinos Center for Biomedical Imaging, Massachusetts General Hospital, Harvard Medical School, Charlestown, MA USA; 3 Department of Psychiatry and Biobehavioral Sciences, David Geffen School of Medicine, University of California Los Angeles, Los Angeles, CA USA; 4 Department of Biostatistics, School of Public Health, University of Alabama at Birmingham, Birmingham, AL USA; 5 Department of Psychiatry, Division of Neurosciences & Clinical Translation, Temerty Faculty of Medicine, University of Toronto, Toronto, ON Canada; 6 Department of Women’s and Children’s Health, Karolinska Institutet, Stockholm, Sweden

**Keywords:** Human behaviour, Psychiatric disorders

## Abstract

In individuals with body dysmorphic disorder (BDD), perceptual appearance distortions may be related to selective attention biases and aberrant visual scanning, contributing to imbalances in global vs. detailed visual processing. Treatments for the core symptom of perceptual distortions are underexplored in BDD; yet understanding their mechanistic effects on brain function is critical for rational treatment development. This study tested a behavioral strategy of visual-attention modification on visual system brain connectivity and eye behaviors. We acquired fMRI data in 37 unmedicated adults with BDD and 30 healthy controls. Participants viewed their faces naturalistically (naturalistic viewing), and holding their gaze on the image center (modulated viewing), monitored with an eye-tracking camera. We analyzed dynamic effective connectivity and visual fixation duration. Modulated viewing resulted in longer mean visual fixation duration compared to during naturalistic viewing, across groups. Further, modulated viewing resulted in stronger connectivity from occipital to parietal dorsal visual stream regions, also evident during the subsequent naturalistic viewing, compared with the initial naturalistic viewing, in BDD. Longer fixation duration was associated with a trend for stronger connectivity during modulated viewing. Those with more severe BDD symptoms had weaker dorsal visual stream connectivity during naturalistic viewing, and those with more negative appearance evaluations had weaker connectivity during modulated viewing. In sum, holding a constant gaze on a non-concerning area of one’s face may confer increased communication in the occipital/parietal dorsal visual stream, facilitating global/holistic visual processing. This effect shows persistence during subsequent naturalistic viewing. Results have implications for perceptual retraining treatment designs.

## Introduction

Body dysmorphic disorder (BDD) is marked by preoccupations with misperceived appearance defects, which sufferers believe render them ugly and deformed, and repetitive behaviors to check or fix one’s appearance. Commonly misperceived appearance features involve the face and head, although any body part can be of concern [1]. The consequences can be profound, with high lifetime prevalence of suicide attempts (25%) [2] and hospitalization (50%) [3]. Twenty-seven to 39% are delusional in their beliefs [4]. BDD is still under-recognized, misdiagnosed, and understudied, although BDD has a high point prevalence of ~2% in the general population [5]. Some neurobiological models to explain vulnerability to BDD have been put forth [6, 7] but a comprehensive understanding of this condition is still emerging.

Disturbances of visual information processing in BDD are likely critical neurobiological contributors to the core psychopathological feature of perceptual distortions of appearance [6, 8]. Our previous neuroimaging studies provide support for this premise. Using own-face [9], other-face [10], and house [11] stimuli as probes in functional magnetic resonance imaging (fMRI) studies, we found abnormally reduced activity in the dorsal visual stream (DVS) when viewing filtered images that contained only low spatial frequency information (i.e., conveying configural/holistic information). This led to the hypothesis that the hyper-scrutiny of miniscule appearance details could be mechanistically related to failing to “see” the appearance feature as an integrated whole, which may reflect an imbalance in global and local processing. This hypothesis has gained support from subsequent imaging and electro-cortical evidence [12, 13]. Adding to the hypothesized model, enhanced ventral visual stream (VVS) processing of high-detail images, and perception of faces as more unattractive when the magnitude of detailed processing increases, were found [12]. Neuropsychological and psychophysical studies testing face and body inversion effects have corroborated the model of imbalance in global vs. local processing [14–19].

Further, selective attention biases potentially contribute to its psychopathological features [20]. This could include aberrant patterns of visual attention, with excessive visual attention paid to perceived appearance defects, which is commonly observed phenomenologically [21]. Studies using eye-tracking in BDD have found biased attention to facial areas deemed flawed, and a scanning pattern characterized by multiple fixations of brief duration [22, 23].

In addition to psychophysical and visual task brain activation studies, functional connectivity studies have also been conducted in BDD [24, 25]. During an others’ face-viewing task, the BDD group demonstrated aberrant connectivity for low spatial frequency images within a face-processing network in the visual and temporal cortices, as well as between the fusiform face area and precuneus/posterior cingulate and insula [24]. During a body-viewing task, individuals with BDD demonstrated reduced dorsal visual network connectivity compared with healthy controls [25]. These studies, testing face-processing and body-processing networks resulted in findings consistent with a model of imbalances in global vs. local visual processing.

Given the phenomenology and the previous research in BDD, some current and proposed treatment approaches [26–28] incorporate visual-attention modifications. Yet, the neural mechanisms underlying aberrant visual attention and how the neurobiological substrates of potential targets are engaged by different visual-attention modification approaches are incompletely understood. A mechanistic understanding is critical for the development of, and ability to iteratively refine, effective clinical treatments.

We therefore designed an experiment to test the neurobiological mechanistic effects of a strategy of visual-attention modification [29]. This strategy requires participants to visually fixate on a centered cross overlaid on their face photo, with eye-tracking monitoring. The purpose is to reduce visual scanning while viewing their face to enhance DVS activity, responsible for global/holistic visual processing, and to suppress VVS activity, responsible for detailed/analytic visual processing.

To examine directional connectivity, we employed dynamic effective connectivity (DEC) modeling [30] to assess connectivity changes from primary visual cortex (V1) to DVS and V1 to VVS over time in different viewing conditions (i.e., unconstrained “natural” viewing of their faces, NatV, and modulated viewing with fixation at a centered cross, ModV). In previous studies we found evidence of hypoactivation in early visual cortical areas such as V1 and early V2 for viewing own faces [9]; as well as hypoactivation in later occipital (V2, V3) and parietal DVS regions, and hyperactivation in temporal fusiform VVS regions for viewing others’ faces [12]. The primary goal was to investigate the effects of visual-attention modulation on the DVS and VVS connectivity during own-face viewing within BDD. As an experimental control, we also investigated connectivity as a result of visual attention modulation in healthy controls. We hypothesized increase fixation duration during ModV compared to NatV across groups, and that fixation duration would correlate with connectivity in DVS. In addition, we hypothesized that ModV would enhance DVS connectivity and suppress VVS connectivity compared to the first NatV in BDD and controls. Further, we hypothesized that during NatV after ModV there would be significant effects on DEC patterns within BDD and controls compared to the first NatV (i.e., a “carryover” effect of the ModV).

## Materials and methods

### Participants

The UCLA Institutional Review Board approved the study. All participants provided informed written consent. Forty-three unmedicated adults with BDD and 35 healthy controls aged 18–40 years were recruited from the community and were enrolled. BDD participants met DSM-5 criteria for BDD, with face concerns. Those with concerns specifically about the region between their eyes were excluded due to the nature of ModV task. BDD participants could have comorbid depressive or anxiety disorders, since they commonly co-occur (See Supplementary Material S1 for exclusion criteria). A power analysis was conducted to address the sample size needed to detect differences in BOLD signal in visual systems for the primary outcomes of interest: the interaction effect between condition and group. For the current study, a sample size of *n* = 30 within each group, after accounting for 12% unusable data due to motion or other artifacts, provided sufficient power of 0.85 to detect small-to-medium effect sizes (*f* = 0.2) for the interaction effect, with *α* = 0.05 (two-tailed). Because this is the first study to test visual modulation in BDD, expected effect sizes were unknown. However, an attentional retraining study in healthy controls that measured changes in brain activity found within-group large effect sizes from 1.29 to 2.80 [31]. This suggests that attention modulation has powerful effects on brain activation, and thus we anticipated that we would be able to detect even more conservative differences with the intervention.

### Clinical assessments

Eligibility was determined through telephone screening followed by a clinical interview with the study physician (JDF). The Mini International Neuropsychiatric Interview (MINI) and BDD Module [32, 33] were administered. The Yale-Brown Obsessive-Compulsive Scale Modified for BDD (BDD-YBOCS) [34], Brown Assessment of Beliefs Scale (BABS) [35], Body Image States Scale (BISS) [36], Montgomery-Åsberg Depression Rating Scale (MADRS) [37], and the Hamilton Anxiety Scale (HAMA) [38] were administered to assess BDD symptoms, insight, evaluative/affective experiences of appearance, depression, and anxiety, respectively (See Supplementary Material S2 for assessment details).

### Task paradigm

**Fig. 1:**
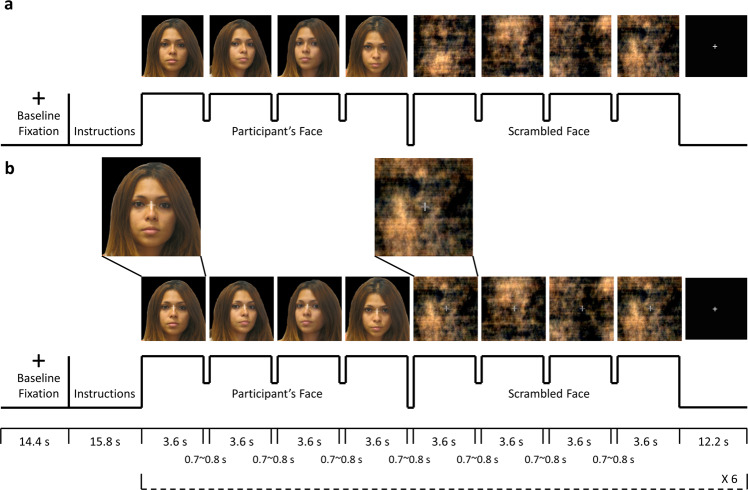
fMRI task paradigm. Four color photos of participants’ own faces at different, standardized angles were captured before the MRI session. A blocked design was used for the presentation of participant’s own face and scrambled face control stimuli for both (**a**) natural viewing and (**b**) visual modulation runs. The first 4 images were participant’s faces at different angles, and the next four images were scrambled faces. Each image was presented for 3.6 s, with a brief gap of 0.7~0.8 s for changing the image. A fixation with duration of 12.2 s was shown after the stimuli. The presentation of participant’s face and scrambled face stimuli was repeated six times in a single run. The stimuli for the visual modulation run (**b**) had a semi-transparent crosshair between the eyes of the participants’ faces and in the center of the scrambled faces. For the visual modulation run, participants were required to maintain their gaze on the crosshair. The rationale was that fixating visual gaze on the crosshair would reduce scanning associated with piecemeal/detailed processing, and enhance holistic/global visual processing. To ensure task compliance for viewing the photos and crosshairs, gaze location was continuously monitored with the camera by the experimenters during the scan. Informed consent was obtained for publication of the image for the volunteer in the figure.

There were two sets of stimuli for the NatV condition: photos of participant’s face and scrambled faces as the control task (Fig. 1a). There were also two sets of stimuli for ModV condition: the same photos overlaid with a semi-transparent crosshair between the eyes, and the scrambled faces with a crosshair (Fig. 1b).

FMRI data were acquired while participants underwent two conditions. During NatV, participants were instructed to view the (unaltered) photos of their face and scrambled images of their face as they normally do. During ModV, they were instructed to view the same images while maintaining attention and eye gaze on the crosshair.

Participants were randomly assigned to one of the two counterbalanced groups for three fMRI runs: NatV-NatV-ModV (NNM) or NatV-ModV-NatV (NMN). They were instructed to press a button every time an image disappeared from the screen to ensure vigilance. Moreover, high-resolution MR-compatible display goggles (VisuaStimXGA, Resonance Technology, Inc.) with a right-side mounted infrared camera was used to present stimuli and record eye gaze. ViewPoint EyeTracker software (Arrington Research, Inc.) sampled pupil location at a rate of 30 Hz. A 9-point calibration was used to normalize the eye gaze position relative to the screen. All values were normalized with respect to mapped *x*-axis and *y*-axis gaze values in a 0.0~1.0 range.

### MRI data acquisition and preprocessing

MRI data were acquired on a 3 T Siemens Prisma scanner. Data preprocessing was done using fMRIPrep 1.4.0 [39]. See Supplementary Material S3–S5 for details of data acquisition and preprocessing, including quality control and motion correction.

### Brain connectivity analysis

**Fig. 2:**
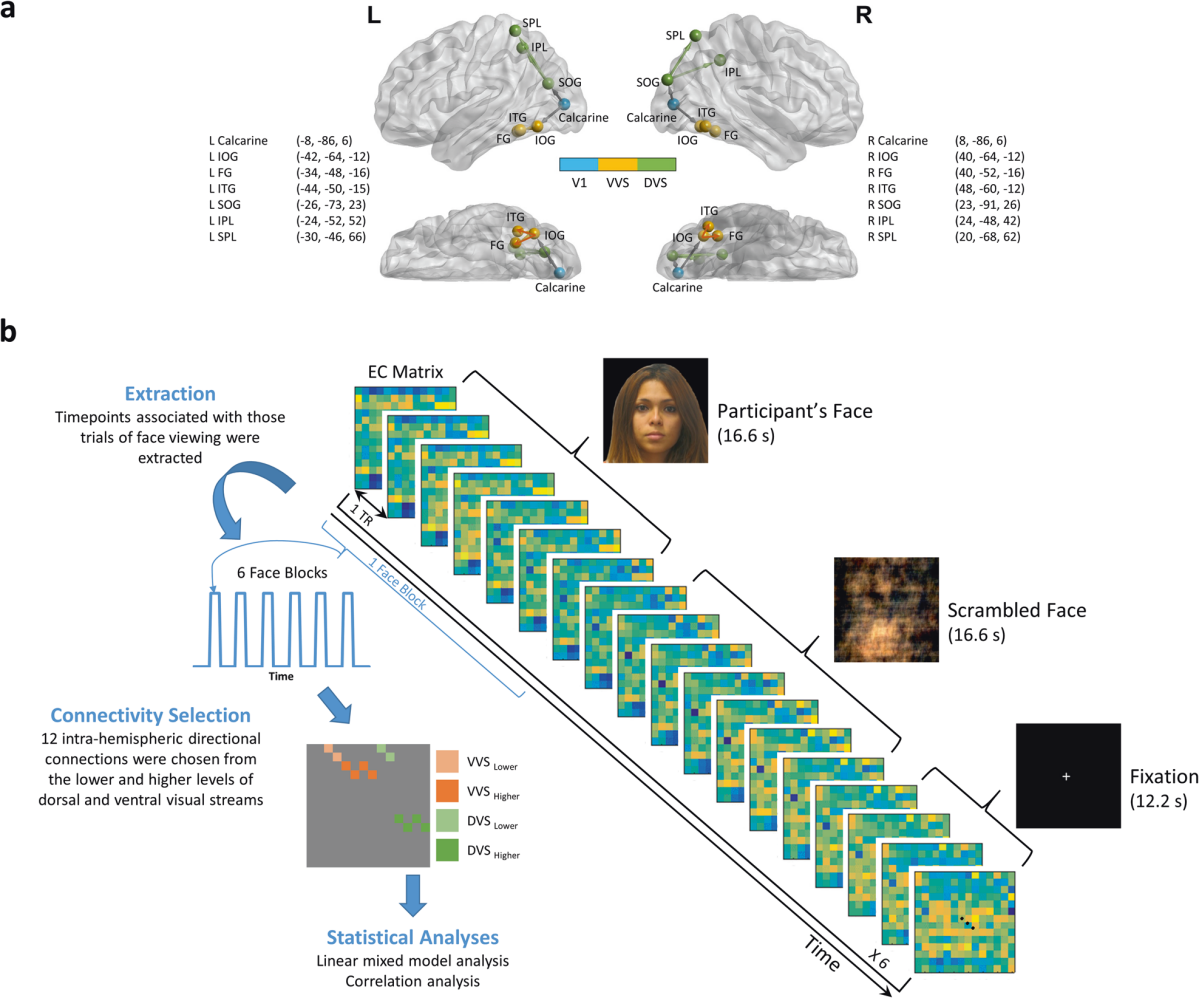
Brain Connectivity Analysis Workflow. **a** Locations of the 14 spherical ROIs used for dynamic effective connectivity analysis, overlaid on a brain surface with lateral and ventral views. These included 2 ROIs in V1 [bilateral calcarine], 6 ROIs in VVS [bilateral inferior occipital gyrus (IOG), fusiform gyrus (FG), and inferior temporal gyrus (ITG)], and 6 ROIs in DVS [bilateral superior occipital gyrus (SOG), inferior parietal lobule (IPL), and superior parietal lobule (SPL)]. The nomenclature is based on Eickhoff-Zilles macro labels from N27, implemented in AFNI. All spheres had a radius of 5 mm and the center-of-mass coordinates obtained from the clusters are *x*, *y* and *z* in the MNI space. This panel was prepared using BrainNet Viewer [70]. **b** Diagram demonstrating the dynamic effective connectivity analysis workflow used to estimate the directional connectivity value from task fMRI data. Effective connectivity (EC) matrices, estimated for each time point, were pooled across task blocks of viewing one’s own face to derive the final EC estimate for each selected connection. *Note: Informed consent was obtained for publication of the volunteer’s photo in the figure.

Fourteen regions-of-interest (ROIs) were derived from the Neurosynth functional meta-analysis in DVS and VVS (Fig. 2). The ROIs in the visual areas were defined using Neurosynth (https://neurosynth.org/) with the search terms including “primary visual”, “ventral visual”, “visual stream”, and “dorsal visual” to obtain maps generated with association tests. Blind-deconvolution [40] was performed on the timeseries extracted from these ROIs to minimize intra-subject variability in hemodynamic response function (HRF) [41], and to improve estimation of effective connectivity [42]. DEC, a time-varying measure of directional connectivity between pairs of ROIs, was computed at each time point using time-varying Granger causality (GC) [30] (Fig. 2). DEC was used because of its ability to estimate causal connectivity across time with a precision of each time point, which helped us capture connectivity only within task blocks of interest. The deconvolved timeseries were fitted into a dynamic multivariate autoregressive (dMVAR) model for estimating DEC between ROIs, which was solved in a Kalman-filter framework. The dMVAR model coefficients vary as a function of time, whose lengths were identical to the number of timepoints in the timeseries. See Supplementary Material S6 for more information. Twelve intra-hemispheric connections were chosen and divided into four categories: (1) VVS_Lower_ *(Calcarine to IOG)*, (2) VVS_Higher_ *(IOG to FG; IOG to ITG)*, (3) DVS_Lower_ *(Calcarine to SOG)*, and (4) DVS_Higher_ *(SOG to IPL; SOG to SPL)*. From these twelve connections, the timepoints associated with those trials of viewing unaltered faces were extracted for subsequent statistical analysis (Fig. 2).

### Gaze analysis

Pupil data were filtered with default settings in the ViewPoint software. Blinks were removed using a blink detection algorithm for low-speed eye-tracking [43]. Missing values of <four consecutive data points (~133 ms) were linearly interpolated, to correct for flicker and loss of contact, considering that saccades typically take 100–130 ms to program [44, 45]. Gaze position values were then smoothed using a Savitzky-Golay filter [46], a simplified least square procedure which is suggested to perform well in low-speed eye-tracking that contains saccade amplitude >5° [47]. Fixations were identified using a velocity threshold algorithm [48] with a velocity threshold of 0.10°/s and a drift threshold of 0.30°/s. Fixations of <100 ms were excluded from the analysis. Mean fixation duration was the main outcome variable to quantify fixation patterns when participants viewed their faces during the face stimuli.

### Statistical analysis

Linear mixed models (LMM) were used to test the primary hypothesis about whether DEC was significantly influenced by experimental factors. Group (BDD or CON), order (NNM or NMN), run (1st or 2nd, or 3rd run), level (Lower or Higher), and their interactions were included in the model as fixed factors, with participant ID as random factor. Pairwise comparisons with Bonferroni correction (*p* < 0.05) were performed afterwards to determine which factors significantly differed from each other. The LMM analysis was done for the separate DVS and VVS hypotheses. Moreover, Pearson correlation was used in exploratory follow-up analyses to determine associations between DEC, symptom severity measures (BDD-YBOCS and BISS), and mean fixation duration. Statistical tests were done using SPSS and R.

## Results

### Sample characteristics

Forty-three BDD participants and 35 controls were eligible and scanned. Among these, we excluded one BDD participant who fell asleep during the experiment and one control as a wrong task paradigm was presented. Moreover, we excluded four BDD and four controls’ data due to excessive motion artifacts, and one BDD’s data due to fMRIPrep errors. Thirty-seven BDD and 30 controls were finally included in the subsequent analyses (Table 1). More details about the sample characteristics can be found in Tables S1 and S2.

**Table 1:** Sample characteristics.

	BDD (*n* = 37)	CON (*n* = 30)	Between-group statistics		
			*χ*^2^	*t*	*p*-value
**Sex (male/female)**	6/31	8/22	1.09		0.30
**Age (years)**	24.8 ± 6.8	23.2 ± 6.8		1.00	0.32
**Symptoms severity**					
HAMA	9.9 ± 7.2	2.5 ± 2.3		5.44	<0.001
MADRS	12.1 ± 9.1	1.1 ± 1.3		6.55	<0.001
BISS	3.6 ± 1.2	6.3 ± 1.1		−9.98	<0.001
BDD-YBOCS	26.8 ± 4.2	NA			
BABS	15.2 ± 4.5	NA			
**Psychiatric comorbidities**					
Major depressive episode	6				
Persistent depressive disorder (dysthymia)	5				
Panic disorder with agoraphobia	2				
Agoraphobia without history of panic disorder	1				
Social phobia	5				
PTSD	2				
Generalized anxiety disorder	11				
No DSM comorbid disorder	18				

*BDD* body dysmorphic disorder, *CON* control, *HAMA* Hamilton Anxiety Scale, *MADRS* Montgomery-Asberg Depression Rating Scale, *BISS* Body Image States Scale, *BDD-YBOCS* Yale-Brown Obsessive-Compulsive Scale Modified for BDD, *BABS* Brown Assessment of Beliefs Scale, *PTSD* Post-traumatic Stress Disorder, *χ*^2^ chi-square test, *t* independent-samples *t*-test.

### Gaze patterns

Across BDD and CON, as hypothesized, mean fixation duration was longer during ModV compared to NatV. Specifically, across groups, mean fixation duration was significantly longer for ModV than the 1st NatV (*p* = 0.023), and the 2nd NatV (*p* = 0.014) for the NNM order. There was a trend for ModV > 2nd NatV (*p* = 0.065) for the NMN order (Fig. S1).

### Brain connectivity patterns

**Fig. 3:**
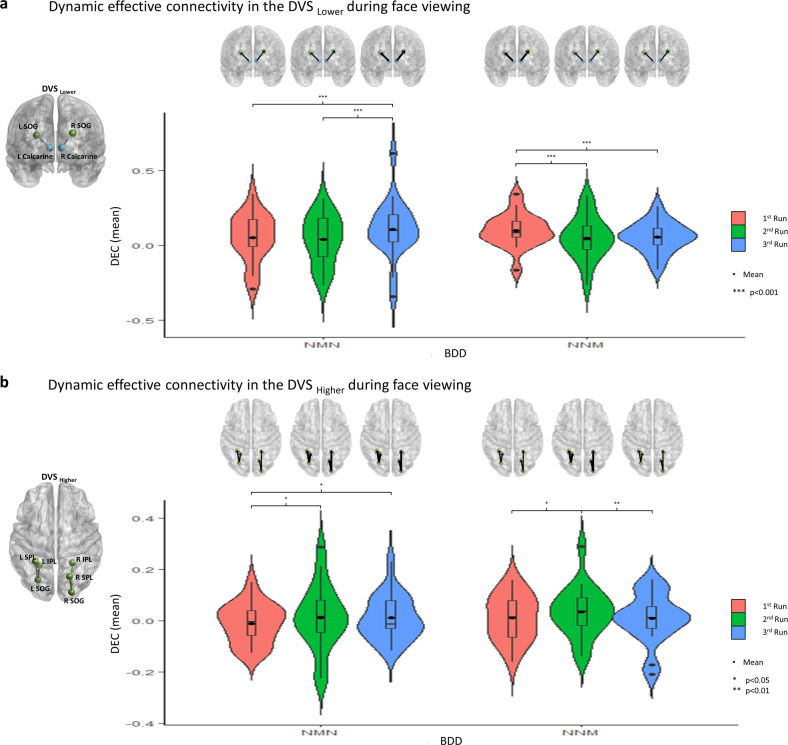
Brain Connectivity in the Dorsal Visual Stream (DVS) in Body Dysmorphic Disorder (BDD) Participants. Means of dynamic effective connectivity for the (**a**) DVS_Lower_ and (**b**) DVS_Higher_ in the BDD group with the NMN and NNM orders. Brain graphs are presented above violin plots in which the thickness of the arrows indicates the relative changes of the mean dynamic effective connectivity values across runs. The participants randomized to the NMN order received natural viewing (N), modulated viewing (M), and then natural viewing (N) as the 1st, 2nd, and 3rd runs; those randomized to the NNM order received natural viewing (N), a second natural viewing (N), and then modulated viewing (M), as the 1st, 2nd, and 3rd runs. The *p*-values were Bonferroni corrected.

In the DVS, from tests of fixed effects, there was a significant four-way interaction between group, order, run and level, F(2,50569) = 3.99, *p* = 0.018. From univariate tests, the simple run effects were significant for DVS_Lower_ in the BDD_NMN_, BDD_NNM_, CON_NMN_, and CON_NNM_ (See Table S3 for statistical values). The simple run effects were also significant for DVS_Higher_ in the BDD_NMN_, BDD_NNM_, CON_NMN_, and CON_NNM_ (See Table S3 for statistical values). Pairwise comparisons were computed between different runs, with a Bonferroni adjustment. For DVS_Lower_, both BDD and controls with the NMN order showed greater DEC during 2nd NatV compared to 1st NatV and ModV (Figs. 3 and S2a), while both BDD and controls with the NNM order exhibited greater DEC during 1st NatV compared to 2nd NatV and ModV (Figs. 3 and S2a). For DVS_Higher_, BDD with the NMN order showed greater DEC during ModV and 2nd NatV compared to 1st NatV, while BDD with the NNM order only showed greater DEC during 2nd NatV compared to 1st NatV. However, controls with the NNM order showed greater DEC during 2nd NatV and ModV compared to 1st NatV, while controls with the NMN order only showed greater DEC during ModV compared to 1st NatV (Figs. 3 and S2a). In sum, ModV resulted in stronger connectivity for DVS_Higher_, and during the NatV that followed it, compared with the first NatV in BDD. All these differences were significant at *p* < 0.05, Bonferroni corrected for three multiple comparisons.

In the VVS, from tests of fixed effects, there was a significant four-way interaction between group, order, run and level, F(2,50572) = 7.68, *p* < 0.001. From univariate tests, the simple run effects were significant for VVS_Lower_ in the BDD_NMN_, BDD_NNM_, CON_NMN_, and CON_NNM_ (See Table S4 for statistical values). The simple run effects were also significant for VVS_Higher_ in the BDD_NMN_, BDD_NNM_, CON_NMN_, and CON_NNM_ (See Table S4 for statistical values). From pairwise comparisons between different runs (*p* < 0.05, Bonferroni corrected for three multiple comparisons), for both VVS_Lower_ and VVS_Higher_, participants with the NNM order showed greater DEC during 1st NatV compared to 2nd NatV. There was no common pattern between BDD and CON with NMN order (Fig. S2b).

### Relationships between brain connectivity and clinical symptoms

**Fig. 4:**
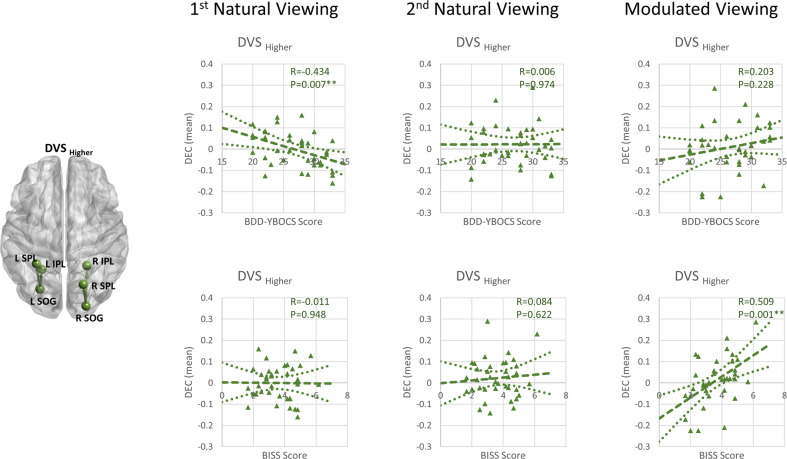
Brain Connectivity and Clinical Symptom Associations. Correlations between mean dynamic effective connectivity (DEC) from occipital to parietal regions in the dorsal visual stream and clinical measures across BDD participants during the first naturalistic viewing, the second naturalistic viewing, and the modulated viewing.

Since a more consistent pattern of DEC changes across the three runs was apparent for the DVS from the results of BDD and CON, the inter-relationships between DEC of the DVS and clinical symptoms (BDD-YBOCS and BISS) were explored with post hoc tests. There was a significantly negative correlation between the DEC for DVS_Higher_ and BDD-YBOCS during the 1st NatV in BDD (*r* = −0.434, *p* = 0.007, uncorrected), and a significantly positive correlation between the DEC for DVS_Higher_ and BISS during the ModV in BDD (*r* = 0.509, *p* = 0.001, uncorrected) (Fig. 4). Those with more severe BDD symptom had weaker DEC for DVS_Higher_ during the initial naturalistic face viewing, while those with poorer body image states also had weaker DEC during the ModV of their own faces. As an exploratory analysis, we also calculated the percentage change in DEC from the 1st NatV to ModV, and a significantly positive correlation was found between the percentage change for DVS_Higher_ and BISS in BDD (*r* = 0.464, *p* = 0.004, uncorrected). The better the body image states, the greater the percentage change in DEC from the 1st NatV to ModV for DVS_Higher_. A larger percentage change in DEC from the 1st NatV to ModV was found in the BDD with the NMN order (mean percentage change: 2.096) compared to the BDD with the NNM order (mean percentage change: −0.213). No significant association was observed between DEC and clinical measures for DVS_Lower_ (Fig. S3).

### Relationships between brain connectivity and visual fixation duration

Testing our hypothesis, for DVS_Higher_, there was a trend for a positive association between DEC and mean fixation duration during the ModV in BDD (*r* = 0.336, *p* = 0.086, uncorrected) (Fig. S4); those with shorter fixation duration tended to have weaker DEC.

### Relationships between visual fixation duration and clinical symptoms

Negative correlations were observed between BDD-YBOCS and mean fixation duration during 1st and 2nd NatV in BDD, although only at trend level for the 2nd NatV (1st NatV: *r* = −0.301, *p* = 0.113, uncorrected; 2nd NatV: *r* = −0.342, *p* = 0.070, uncorrected) (Fig. S5); BDD individuals with more severe BDD symptom tended to have shorter fixation duration during NatV.

## Discussion

The goal of this study was to understand how brain connectivity and visual fixation patterns in those with BDD when viewing one’s face—the primary area of appearance concern for most—change under conditions of modulated visual attention. We specifically investigated how brain connectivity and visual fixation are influenced by visual-attention modulation, and if there are subsequent “carryover” effects when viewing one’s face naturalistically after visual-attention modulation. Visual-attention modulation resulted in stronger connectivity from occipital to parietal DVS regions, and during the naturalistic face viewing that followed it, compared with the first naturalistic viewing in BDD. Longer fixation duration was associated with a trend for stronger connectivity during modulated viewing. Further, those with more severe BDD symptoms had weaker connectivity during the first naturalistic viewing, and those with more negative appearance evaluations had weaker connectivity during visual-attention modulation. There was a trend for those with more severe BDD symptoms to have shorter fixation duration during the second naturalistic viewing. We did not confirm our hypothesis that modulated viewing resulted in decreased VVS connectivity. Nevertheless, these results largely follow our model-based predictions that those with more severe BDD symptoms would fixate on their faces for a shorter period, and longer fixation during visual-attention modulation would be associated with enhanced communication in systems responsible for global visual processing. These findings shed light on the inter-relationships between brain connectivity, eye behaviors and clinical symptomatology. Importantly, they demonstrate the mechanistic effects of a brief attention modulation intervention of holding gaze constant on brain connectivity and visual fixation.

There are several important implications of these findings that could impact future translational research involving perceptual retraining in BDD. These results provide early evidence that changing eye-gaze behaviors might change the balance of global vs. local processing mediated by the DVS. This has been suggested in ongoing [49] and planned [50] treatment protocols. The observation of maintained increase in DVS connectivity during the naturalistic viewing following the brief period of visual-attention modulation (fixating on a non-concerning region of the face) suggests the possibility of a persistent DVS effect that may enhance global/holistic processing. A similar phenomenon was demonstrated in a study in which exposure to a Navon visual stimulus [51]—a large letter made of smaller letters that was presented in a way to promote a global bias—induced global processing and temporarily reversed visual processing biases in individuals with great body image concerns [52].

In the current study, there was a trend for occipital to parietal DVS connectivity magnitude to scale with the fixation duration during visual-attention modulation; those with longer fixation duration had stronger connectivity. Although there was no significant increase on average in visual fixation duration from visual-attention modulation to the following naturalistic viewing, individuals with longer fixation duration, which may have persisted from the visual-attention modulation to the following naturalistic viewing, could have experienced persistently enhanced DVS connectivity. In this second naturalistic viewing, there were no explicit instructions other than to view their face as they normally do, so changes in gaze patterns were likely implicit, although some participants might have willfully tried to reduce scanning during this period.

Both BDD and controls showed longer fixation duration during visual-attention modulation compared with naturalistic viewing. This was expected due to the task that required them to fixate their gaze on a centered cross. It also demonstrated overall compliance with the instructions. In general, eye-movement parameters, including fixation duration and saccade amplitude, can be used to characterize distinct modes of visual processing [53], which may indicate differential involvement of dorsal and ventral systems in saccade planning and information processing. Although we did not directly quantify saccades due to limitations of the data from the goggle-mounted eye-tracker camera, fixation on a crosshair would be expected to be accompanied by fewer saccades than during naturalistic viewing. Saccades have been found to suppress low spatial frequency (dorsal pathway) contrast sensitivity [54], suggesting a reduction of global/configural processing. Moreover, the frontal eye fields for controlling visual attention and eye movements have dense connections with the occipitoparietal network (in the DVS) [55], such that reduction of eye scanning (also reduced occurrence of saccades) would be expected to enhance DVS activity. Our findings corroborate this model, that longer fixation duration associates (at trend level) with stronger effective connectivity in the DVS. Thus, potential changes in attentional allocation in conjunction with eye gaze behavior may have a modulatory effect on the DVS, especially when they were instructed to fixate on the crosshair that was evident during the visual-attention modulation.

Alternatively, previous studies of eye behaviors describe a “pre-attentive” mode, in which scanning eye movements are predominant with brief fixations and large saccades, while in an “attentive” mode, long fixations and small saccades are present, leading to detailed inspection [53, 56, 57]. In theory, pre-attentive scanning behavior could reflect dorsal pathway processing, while attentive inspection behavior could reflect ventral pathway processing [53, 58]. However, it is important to note that the studies characterizing these viewing modes were based on scene viewing and may not apply to face processing; scenes are highly variable and novel, yet humans have high expertise and specialized visual “templates” for faces. Further, those studies did not specifically examine dynamic connectivity patterns as in this study.

The current study also demonstrated that those with more severe BDD symptoms had a trend for shorter fixation duration during the second naturalistic viewing. Previous eye-tracking studies in BDD have shown aberrant scan-paths when viewing stimuli such as faces. These scan-paths are generally characterized by either a “focused” pattern—paying attention to areas of concern, or an “avoidant” pattern—avoiding perceived defects [22, 23, 59, 60]. In these studies, BDD participants showed aberrant eye movements, including enhanced selective visual attention to imagined defects, overfocus on negative attributes, or atypical scanning behaviors with more blinks, fewer fixations, and less visual attention paid to prominent facial features. Abnormalities in face-processing are particularly evident in BDD when viewing own-faces and faces showing negative or neutral emotional expressions [59, 60]. In a study examining attention to attractive vs. unattractive parts of one’s own and other’s faces in participants with BDD, bulimia nervosa, and controls, participants with BDD and bulimia nervosa spent less time looking at attractive parts of their own face than controls, yet more time looking at attractive parts than unattractive parts of other’s faces [61]. This suggests ignoring of positive aspects of one’s face in BDD, and/or upward social comparison, either or both of which could account for the increase in negative emotions observed in BDD after face viewing. In this study, although only at trend level, shorter fixation duration during NatV, in those with more severe BDD symptoms, suggests multiple short-duration fixation patterns interspersed with an increased number of saccades for scanning multiple facial details. This could reflect heightened attention to multiple perceived appearance flaws, or, alternatively, an unwillingness to fixate on any one area of their own faces more than briefly due to a triggering of negative emotions.

The observation of a negative association between BDD symptom severity and DVS connectivity during the initial naturalistic viewing is in line with findings of our previous studies. We demonstrated previously that BDD exhibits hypoactivity in the DVS when viewing low spatial frequency images [9–13], and weaker connectivity in parietal network during a body-viewing task [25], compared to controls. In the current study, we also demonstrate that worse body image self-evaluation (BISS scores) is associated with weaker DVS connectivity during visual-attention modulation, and, further, that lower percentage changes in DVS connectivity from the first naturalistic viewing to visual-attention modulation occurred in those with worse body image.

It is important to note that body image evaluation, as measured by the BISS, may represent an experiential construct that is partially overlapping (but non-identical) to appearance-related preoccupations and repetitive/compulsive behaviors in individuals with BDD, as measured by the BDD-YBOCS. Body image is conceptualized as comprising feelings, thoughts, behaviors, and evaluations associated with one’s body [62], and is a subjective picture of one’s body/appearance that may differ from how one’s body/appearance is perceived by others [63, 64]. Body image disturbance is an important component of several psychiatric diseases that involve appearance concerns, including BDD, anorexia nervosa, and bulimia nervosa [65]. Body image disturbance can be conceptualized to include perceptual disturbance, involving failure to evaluate accurately one’s body size or other appearance features, and conceptual disturbance, involving negative feelings and cognitions of attitudinal or affective perception of one’s body. Body image disturbance can also manifest as behavioral disturbance, including body avoidance, body checking, or dieting [66]. Following from this, it was unsurprising that the BDD participants in our sample exhibited significantly lower BISS scores compared to controls, reflecting current body dissatisfaction, and there was a weakly negative correlation (*r* = −0.184, *p* = 0.275) between BISS and BDD-YBOCS scores. Like the interpretation of the negative association between BDD symptom severity and DVS connectivity, BDD participants with poorer body image also may have worse global visual processing during visual attention-modulation. Moreover, those with poorer body image had less enhancement of connectivity in the DVS during visual-attention modulation compared with the first naturalistic viewing. This suggests that this mode of visual processing may be more resistant to this intervention in those with worse body image. Further, lower DEC changes from the first naturalistic viewing to visual-attention modulation in those who had two runs of naturalistic viewing before the visual-attention modulation (the NNM order as opposed to the NMN order) suggest that longer periods of naturalistic viewing could also contribute to more resistance to a visual-attention modulation intervention.

There are several limitations to consider. The study population underrepresents the proportion of males with BDD in the general population [67, 68], thus findings may not generalize. Another limitation is that we did not assess participants’ emotional states during face viewing (in the interest of not interrupting natural processes involved in face viewing that might be disrupted by self-reflection). Thus, we could not investigate how degree of emotional arousal, such as anxiety [69], affects visual system activity. Moreover, we were unable to use areas-of-interest on the face photographs due to limitations in positional stability of the goggle-mounted eye-tracking camera that otherwise might be informative about viewing patterns of areas of concern during naturalistic viewing after visual-attention modulation.

In conclusion, these findings provide evidence that holding gaze constant on a non-concerning area results in enhanced dynamic connectivity from occipital to parietal DVS regions when individuals with BDD view their face. After this visual-attention modulation, this effect persists when viewing faces naturalistically. This provides a behavioral and neural mechanistic proof-of-concept that visual-attention modulation may enhance global/configural visual processing and have a subsequent, persistent carryover effect during subsequent natural viewing of one’s face. The potential clinical relevance is underscored by the observed neural-behavioral phenotype associations; those with more severe BDD symptoms had weaker DVS connectivity during the first naturalistic face viewing, and those with poorer body image had weaker DVS connectivity during modulated viewing. Visual-attention modulation thus holds promise for future translational studies of perceptual retraining for BDD.

## Supplementary information

Supplementary materials

## Data Availability

The computer code used to generate the results reported in this study are available from the corresponding author on request.
